# Functional Assessment *In Vivo* of the Mouse Homolog of the Human Ala-9-Ser NHE6 Variant

**DOI:** 10.1523/ENEURO.0046-19.2019

**Published:** 2019-11-24

**Authors:** Qing Ouyang, Lena Joesch-Cohen, Sasmita Mishra, Hasib A. Riaz, Michael Schmidt, Eric M. Morrow

**Affiliations:** 1 Department of Molecular Biology, Cell Biology and Biochemistry, Brown University, Providence, RI 02912; 2 Center for Translational Neuroscience, Robert J. and Nancy D. Carney Institute for Brain Science and Brown Institute for Translational Science, Brown University, Providence, RI 02912; 3 Developmental Disorders Genetics Research Program, Emma Pendleton Bradley Hospital, East Providence, RI 02915; 4 Hassenfeld Child Health Innovation Institute, Brown University, Providence, RI 02912; 5 Department of Psychiatry and Human Behavior, The Warren Alpert Medical School of Brown University, Providence, RI 02912

**Keywords:** Christianson syndrome, endosomes, genetics, SLC9A6, variant of unknown significance

## Abstract

Christianson syndrome (CS) is an X-linked neurogenetic disorder resulting from loss-of-function (LoF) mutations in *SLC9A6*, which encodes the endosomal Na^+^/H^+^ exchanger 6 (NHE6). NHE6 regulates proton efflux from endosomes and, thus, participates in regulating cargo processing and trafficking. LoF mutations in NHE6 cause aberrant acidification of endosomes. While CS arises in males generally due to clear LoF mutations, other potentially hypomorphic variants have emerged, yet most of these variants have not been evaluated for functional effects, particularly *in vivo*. Here we characterize an *SLC9A6* variant that has been previously reported in patients, yet now also appears in exome datasets of largely control individuals—c.25G>T, p.A9S. By heterologous expression in cell lines, we show that human NHE6A9S is expressed and localizes in a manner comparable to control NHE6. By genome editing, we generated the equivalent NHE6 mutation in mouse—p.A11S—and determined that male NHE6A11S mice have normal brain size at 6 months of age and do not show cerebellar degeneration or defective neuronal arborization. Neurons from male NHE6A11S mice also did not demonstrate an abnormality in intraendosomal pH compared with controls. These findings are in contrast to findings in NHE6-null mice previously reported and indicate that the NHE6A11S variant functions at a level equivalent to control NHE6 for many of the assays performed. These data stand in support of the population genetic data, which are also evaluated here, indicating that the A9S variant is unlikely to confer disease susceptibility with high penetrance.

## Significance Statement

Loss-of-function mutations in *SLC9A6*, encoding Na^+^/H^+^ exchanger 6 (NHE6), cause Christianson syndrome. Also, missense variants of unknown consequences have emerged. Functional evaluation of these mutations contributes to an understanding of the medical relevance and cellular effects of these variants. We show that human NHE6A9S protein is synthesized and localizes normally. Mice genome edited to the equivalent variant—NHE6A11S—did not show a reduction in brain size or defects in neuronal arborization. Mutant NHE6A11S neurons also did not demonstrate a significant decrease in luminal pH of endosomes. These findings are in contrast to findings in NHE6-null mice previously reported. Combined with the population genetic data, our data indicate that the NHE6A9S variant is unlikely to confer disease susceptibility with high penetrance.

## Introduction

Christianson syndrome (CS) is an X-linked neurogenetic disorder first described in a South African family in 1999 (Christianson et al., 1999). The core clinical features of CS include moderate to severe intellectual disability, nonverbal status, epilepsy, truncal ataxia, postnatal microcephaly, and hyperkinetic behavior (Christianson et al., 1999; Pescosolido et al., 2014). Secondary clinical features include eye movement abnormalities, motor regression, cerebellar atrophy, low body weight, and a happy demeanor resembling Angelman syndrome (AS; Christianson et al., 1999; Gilfillan et al., 2008; Schroer et al., 2010; Mignot et al., 2013; Pescosolido et al., 2014). The genetic basis of CS is loss-of-function (LoF) mutations in the X-linked gene *SLC9A6*, which encodes the endosomal Na^+^/H^+^ exchanger 6 (NHE6; Gilfillan et al., 2008; Garbern et al., 2010; Takahashi et al., 2011; Tzschach et al., 2011; Mignot et al., 2013; Riess et al., 2013; Pescosolido et al., 2014; Zanni et al., 2014; Masurel-Paulet et al., 2016). Systematic sequencing of X-chromosome genes has indicated that CS is among the most common X-linked developmental brain disorders (Tarpey et al., 2009).

NHE6 belongs to the SLC9A gene family. NHE6 mainly localizes to early, recycling, and late endosomes, while also transiently localizing to the plasma membrane (Brett et al., 2002; Nakamura et al., 2005; Ohgaki et al., 2008, 2010; Ouyang et al., 2013). By moving H^+^ out of the organellar lumen in exchange for Na^+^ or K^+^, organellar NHEs such as NHE6 participate in regulating luminal pH, which is an essential aspect of the processing and trafficking of intracellular cargo (Orlowski and Grinstein, 2004; Ohgaki et al., 2011; Kondapalli et al., 2014). In neurons, NHE6 is found in growing axons and dendrites and at branch points. It also colocalizes with presynaptic and postsynaptic markers (Deane et al., 2013; Ouyang et al., 2013), indicating that NHE6 is involved in many cellular processes critical to normal nervous system development and function.

More than 30 distinct mutations in *SLC9A6* have been identified in CS patients (Gilfillan et al., 2008; Garbern et al., 2010; Schroer et al., 2010; Takahashi et al., 2011; Mignot et al., 2013; Schuurs-Hoeijmakers et al., 2013; Bosemani et al., 2014; Pescosolido et al., 2014; Zanni et al., 2014; Masurel-Paulet et al., 2016; Trump et al., 2016; Padmanabha et al., 2017; Kerner-Rossi et al., 2018; Weitensteiner et al., 2018). Most of the mutations in *SLC9A6* are nonsense mutations thought to result in truncation of the NHE6 protein or destruction of *NHE6* mRNA by nonsense-mediated decay; thereby, the majority of mutations are LoF mutations (Kondapalli et al., 2014; Pescosolido et al., 2014). As supported by mouse studies, the loss of NHE6 function results in overacidification of the endosomal compartment, attenuated TrkB signaling, decreased neuronal arborization and circuit strength (Ouyang et al., 2013), and, potentially, disruption of neurotransmitter receptor trafficking (Deane et al., 2013).

Other types of mutations and variants identified in *SLC9A6* include in-frame deletions, missense mutations, and splicing mutations (Fichou et al., 2009; Riess et al., 2013; Pescosolido et al., 2014; Zanni et al., 2014; Ilie et al., 2016; Masurel-Paulet et al., 2016; Padmanabha et al., 2017; Weitensteiner et al., 2018). A limited number of these mutations have been assessed *in vitro* (e.g., expression in heterologous cell lines), with results indicating that the NHE6-mutant proteins are unstable and do not undergo appropriate protein maturation and that their expression leads to defects in the functioning and survival of cells (Gilfillan et al., 2008; Roxrud et al., 2009; Ilie et al., 2014, 2016, 2019). Molecular and cellular characterization of *SLC9A6* variants such as missense variants will contribute to our understanding of their medical significance, as well as broaden our understanding of the function of NHE6.

The purpose of the study presented here was to evaluate a missense mutation of *SLC9A6* reported by Fichou et al. (2009) wherein a c.25G>T mutation in *SLC9A6* exon 1 results in an alanine-to-serine substitution at position 9 of NHE6 (p.A9S). This mutation was identified in a cohort of male patients with AS-like features. Subsequently, however, the NHE6A9S variant has been found in large, putative control exome sequencing databases, calling into question the medical significance and disease-causing role of this variant. We provide results here based in expression of wild-type and mutant NHE6 in cultured cells and analysis of a mouse model with an equivalent mutation (p.A11S) indicating that the alanine-to-serine mutant NHE6 performs in a manner largely similar to wild-type NHE6 with respect to the tested functional measures. Our data, combined with the human population genetic data as evaluated here, provide support for the interpretation that this human gene variant is unlikely to confer susceptibility to disease with high penetrance. As such, these results may assist in interpretation of genetic diagnostic information.

## Materials and Methods

### Analysis of the population frequency and sex skew of the A9S variant in SLC9A6

The total number of male (AN_male) and female (AN_female) chromosomes covered at the location of the A9S variant in *SLC9A6*, along with the number of mutant female alleles (AC_female), mutant male alleles (AC_male), and heterozygotes (AC_het), was extracted from the Exome Aggregation Consortium (ExAC) database (http://exac.broadinstitute.org/; Lek et al., 2016; Table 1). Based on the information in Table 1, the observed number of individuals with each possible genotype for this locus was calculated using the following equations:

M (total number of male samples covered at this location)=AN_male,

F (total number of female samples covered at this location)=AN_female/2,

X(mut)/Y=AC_male,

X(wt)/Y=M−AC_male,

X(mut)/X(wt)=AC_het,

X(mut)/X(mut)=(AC_female − AC_het)/2,

and

X(wt)/X(wt)= F −(X(mut)/X(mut)+ X(mut)/X(wt)).

**Table 1:** Values for the A9S variant of SLC9A6 reported in the ExAC database

AN_female	13,626
AN_male	1232
AC_female	92
AC_male	22
AC_het	92
*q*	0.00767

AC, Total number of chromosomes with variant allele; AN, total number of chromosomes able to be called regardless of whether reference or variant allele; *q*, frequency of variant allele.

The results of the above calculations are shown in Table 2 under the “Observed” column.

**Table 2:** Observed and expected values for different genotypes of the A9S variant of SLC9A6

Genotype	Observed	Expected
*X*(wt)/*X*(wt) = homozygous wild-type female	6721	6709
*X*(wt)/*X*(mut) = heterozygous female	92	104
*X*(mut)/*X*(mut) = homozygous mutant female	0	0
*X*(wt)/*Y* = hemizygous wild-type male	1210	1223
*X*(mut)/*Y* = hemizygous mutant male	22	9

mut, Mutant; wt, wild-type.

To calculate the expected number of individuals of each genotype, Hardy–Weinberg equilibrium was assumed, with *q* (the frequency of the mutant allele) given by ExAC, as reported in Table 1. This frequency value was used in conjunction with the *F* and *M* values defined above and the following equations:

p (frequency of wild-type allele)=1 – q,

X(wt)/Y=M*p,

X(mut)/Y=M*q,

X(mut)/X(mut)=F*(q^2),

X(mut)/X(wt)=F*2*p*q,

and

X(wt)/X(wt)=F*(p^2).

The results of the above calculations are shown in Table 2 under the “Expected” column.

### Cell line and mouse lines

For experiments involving use of cultured cells, the HeLa cell line was used (CCL-2, ATCC; RRID:CVCL_0030). For studies in the mouse, male mice from the following two *Nhe6* mouse lines were used: a mouse model of the human A9S variant (A11S in mouse) of NHE6; and a new NHE6-null mouse model due to a 4 bp CAAG deletion in exon 15 that causes a frameshift and subsequent generation of a premature stop codon (ChrX: g.56658353-56658356, c.1825-1828, GRCm38; NCBI Reference Sequence: NC_000086; Ensembl Transcript: ENSMUST00000077741.11). These mouse lines were generated using CRISPR/Cas9-mediated genome editing (Mouse Transgenic and Gene Targeting Facility of Brown University) and are described for the first time herein. Both mouse lines are on the C57BL/6N mouse background. The targeting of constructs and the presence of mutations were confirmed by PCR genotyping and Sanger sequencing. Western blotting was used to confirm protein expression or lack thereof (see Fig. 2). All experiments involving live animals were conducted in accordance with the U.S. National Institutes of Health *Guide for the Care and Use of Laboratory Animals* (National Research Council of the National Academies, 2011) under a protocol approved by the Brown University Animal Care and Use Committee.

### DNA constructs

The human *NHE6* gene (GenBank: NM_006359) was cloned into a pReceiver-M07 mammalian expression vector to generate a C-terminal HA-tagged pNHE6–HA construct. The pNHE6–HA construct was then used, together with the QuickChange site-directed mutagenesis kit (Stratagene), to generate a construct encoding for an HA-tagged version of the A9S variant of human NHE6 (NHE6A9S-HA). Mutagenesis was performed according to the manufacturer protocol and using the following overlapping primers: (5′-GCGGAGGGGTGACCGCCGCCAGC-3′) and (5′-GCTGGCGGCGGTCACCCCTCCGC-3′).

### PCR genotyping

To confirm the G > T mutation targeting strategy in the NHE6A11S-mutant mouse model, PCR genotyping was performed using mouse tail lysates and the following primers: 141_Slc9a6_ex1F1 (5′-CTGTAGGTGGGTAGACAAGCC-3′) and 147_Slc9a6_ex1R4 (5′-TTCTCGGACACGATCTCCTC-3′). Target bands of 402 bp were gel purified, followed by Sanger sequencing to determine genotype. The 4 bp deletion in the NHE6-null mouse model was confirmed by PCR genotyping using the following primers: 522_Slc9a6_ex15uF3 (5′-CAGTAGAGCACTTGTAGAGCAC-3′) and 555_Slc9a6_ex15dR5 (5′-CAGAAGCCTAACCCATAGAAC-3′). Target bands of 406 bp were gel purified, followed by Sanger sequencing to determine genotype.

### Western blotting

Mouse whole-brain lysates or HeLa cell lysates were prepared and subsequently heated at 70°C for 10 min in 4× NuPage LDS sample buffer (catalog #NP0007, Invitrogen) with 10× NuPage sample reducing agent (catalog #NP0009, Invitrogen). Samples were then subjected to SDS-PAGE in a 4–12% polyacrylamide gel and transferred to nitrocellulose membranes according to standard procedures. Western blotting was performed as follows. The membrane was blocked for 1 h at room temperature using Odyssey TBS blocking buffer (catalog #927-50000, LI-COR), incubated overnight at 4°C in primary antibody diluted in TBS blocking buffer, rinsed 3 × 10 min with TBS-Tween, incubated for 1 h at room temperature in secondary antibody diluted in TBS blocking buffer, rinsed 3 × 10 min with TBS-Tween, and scanned with an Odyssey CLx Infrared Imaging System (LI-COR; RRID:SCR_014579). The primary antibodies used were rabbit anti-NHE6 (048; 1:1000 working dilution; Ouyang et al., 2013), mouse anti-α-tubulin (1:4000 working dilution; catalog #T6074, Sigma-Aldrich; RRID:AB_477582), and rabbit anti-HA (1:1000 working dilution; catalog #3724s, Cell Signaling Technology; RRID:AB_1549585). The secondary antibodies used were goat anti-rabbit IRDye680 (1:20,000 working dilution; catalog #925-68071, LI-COR; RRID:AB_2721181) and goat anti-mouse IRDye800 (1:20,000 working dilution; catalog #925-32210, LI-COR; RRID:AB_2687825).

### Immunocytochemistry

For cultured HeLa cells, cells were first rinsed twice with 1× PBS. Cells were then fixed in 4% (w/v) paraformaldehyde (PFA) for 15 min and permeabilized with 0.25% (w/v) Triton X-100 in 1× PBS for 15 min at room temperature. Cells were then blocked with 10% normal goat serum in 1× PBS + 0.1% Triton X-100 (PBST) for 1 h at room temperature followed by incubation overnight at 4°C with primary antibodies diluted in PBST containing 2% normal goat serum. The primary antibodies used were rabbit anti-NHE6 (048; Ouyang et al., 2013) and mouse anti-HA (catalog #11583816001, Roche; RRID:AB_514505). After rinsing 3 × 5 min in PBST, cells were incubated for 1 h at room temperature with secondary antibodies diluted in PBST containing 2% normal goat serum. The secondary antibodies used were goat anti-rabbit Alexa Fluor 488 (1:800 working dilution; catalog #A-27034, Thermo Fisher Scientific; RRID:AB_2536097) and goat anti-mouse Alexa Fluor 594 (1:800 working dilution; catalog #A-11032, Thermo Fisher Scientific; RRID:AB_2534091). Additionally, nuclei were counterstained with DAPI (1:1000 working dilution; Thermo Fisher Scientific) at the same time as incubation with secondary antibody. Cells were again rinsed 3 × 5 min in PBST and then mounted in Fluoromount-G (SouthernBiotech).

For hippocampal cultures, wild-type and NHE6A11S-mutant neurons were first starved with Earle’s balanced salt solution (EBSS) for 30 min, and then cells were incubated with 25 μg/ml Alexa Fluor 568-conjugated transferrin (catalog #T23365, Thermo Fisher Scientific) for 10 min. After washing three times with 1× PBS, cells were fixed with 4% PFA and immunostained using rabbit anti-NHE6 as the primary antibody (048; Ouyang et al., 2013) and goat anti-rabbit Alexa Fluor 488 as the secondary antibody (1:800 working dilution; catalog #A-27034, Thermo Fisher Scientific; RRID:AB_2536097). Nuclei were counterstained with DAPI (1:1000 working dilution; Thermo Fisher Scientific).

Confocal microscopy images were collected using a Zeiss LSM 710 confocal microscope. Structured illumination microscopy (SIM) images were collected using a DeltaVision OMX SR microscope. Z-series images were collected using a 60× oil objective (refractive index immersion oil 1.516) and structured illumination (SI) light path under sequential acquisition mode. Z-series images were processed by performing OMX SI reconstruction, alignment, and maximal projection sequentially using softWoRx software. Images were analyzed using ImageJ software (NIH).

### Tissue preparation

Mice were anesthetized with Beuthanasia-D and transcardially perfused with phosphate-buffered 4% PFA. Brains were removed from skulls, stored in 4% PFA overnight at 4°C, sequentially immerged in 10%, 20%, and 30% sucrose solutions at 4°C for 1 d per solution of a specific percentage of sucrose, embedded in optimal cutting temperature compound, and stored at −80°C.

### Brain size measurement

Matched male littermate pairs collected at 6 months of age were transcardially perfused with 4% PFA and imaged using a Leica MZ16F dissecting microscope. The areas of the cortical hemispheres, midbrain, and cerebellum were measured using ImageJ software (NIH).

### Histology and immunohistochemistry

For cerebellar degeneration analysis, 30 μm sagittal sections were collected using a Leica Cryostat. To perform immunohistochemistry, sections were first prewetted with 1× PBS for 15 min. Sections were then postfixed with 4% PFA for 15 min, permeabilized with 0.25% (w/v) Triton X-100 in 1× PBS for 15 min, blocked with 10% normal goat serum in PBST for 2 h at room temperature, and incubated overnight at 4°C with mouse anti-calbindin antibody diluted in PBST containing 2% normal goat serum (1:1000 working dilution; Swant 300, Swant; RRID:AB_10000347). After washing the sections extensively with PBST, they were incubated with Alexa Fluor 568-conjugated goat anti-mouse secondary antibody diluted in PBST containing 2% normal goat serum (1:800 working dilution; catalog #A-11004, Thermo Fisher Scientific; RRID:AB_2534072) for 2 h at room temperature. The sections were then washed three times with PBST and mounted using Fluoromount-G (SouthernBiotech).

### Endosomal pH, transferrin distribution, and endosome size analyses in mouse hippocampal neurons using the Opera Phenix High-Content Screening System

Isolated hippocampal neurons were seeded at a density of 28,000 cells/well in CellCarrier-96 Ultra Microplates (clear bottom black; PerkinElmer). For each pup, three to four wells of cells were seeded and analyzed, which served as a means of experimental replication. At 5 d *in vitro* (DIV), cells were first starved in EBSS at 37°C for 30 min. EBSS was then replaced with regular Neurobasal-A media containing 33 μg/ml fluorescein isothiocyanate (FITC)-conjugated transferrin (catalog #T2871, Thermo Fisher Scientific), which is pH sensitive, and 33 μg/ml Alexa Fluor 546-conjugated transferrin (catalog #T23364, Thermo Fisher Scientific), which is pH insensitive. Nuclei were simultaneously counterstained with DAPI to facilitate data analysis. After a 10 min incubation, the cells were washed twice with warm PBS, placed in media lacking phenol red, and imaged live under 60× water objective using an Opera Phenix High-Content Screening System (PerkinElmer). Twenty images were taken per well. To generate the standard curve for use in determining endosomal pH, similar procedures were followed; however, neurons were imaged in standard buffer solutions containing the following: 125 mm KCl, 25 mm NaCl, 10 μm monensin, and either 25 mm HEPES, for a standard pH value of 7.0, or 25 mm MES, for standard pH values of 6.5, 6.0, and 5.5, and adjusted to a final pH using 1N NaOH or 1N HCl.

Confocal images were analyzed using Harmony software (PerkinElmer). The DAPI channel was used to identify nuclei and the somal region using the Find Nuclei and Find Cytoplasm building blocks. Detected nuclei were used to count cells in the assays, and a minimum nuclear area threshold criterion was applied to select for live cells. Transferrin-labeled endosomes in soma and neurites were identified using the Find Spots building block. To specify which kind of spots should be detected, the following parameters were adjusted: (1) specify region (soma or neurites) for the spots; and (2) specify the filter criteria to select spots by their fluorescence intensity and area. A spot area cutoff of 15–90 square pixels was chosen to represent endosomes, and the fluorescence intensity of each fluorophore was measured within endosomes. Also, data regarding the number of transferrin-labeled endosomes in neurites and soma and the live cell number were collected. To determine endosomal pH, the ratio of FITC fluorescence intensity to Alexa Fluor 546 fluorescence intensity was calculated. For the standard curve, the ratio was plotted against pH values; for experimental measures, the ratio was extrapolated to the standard curve. To assess transferrin distribution, endosome number and localization (soma or neurite) were determined in live cells based on the Alexa Fluor 546-conjugated transferrin signal. To compare the sizes of endosomes, area values for the spots analyzed were exported and square pixels were converted to square micrometers.

### Cerebellum image quantification

The “Sagittal Atlas” from the Allen Mouse Brain Atlas was used to determine sagittal sections of the cerebellum to analyze (Allen Institute for Brain Science, 2004). Sections selected for mid-sagittal analyses most closely resembled Position 195 and Position 201 from the Sagittal Atlas (http://mouse.brain-map.org/experiment/thumbnails/100042147?image_type=atlas). Images of the cerebellum were acquired as *z*-stacks comprising 18 × 1.63 μm slices using an Olympus FV3000 confocal microscope equipped with a 20× objective. Individual images were stitched into a whole image. Purkinje cell (PC) body density was calculated as the number of PC bodies divided by the length of the PC layer measured. All images were analyzed using ImageJ software (NIH).

### Continuous live-cell imaging and neurite outgrowth analysis

Continuous live-cell imaging was performed using an IncuCyte S3 Live-Cell Analysis System (Essen BioScience). For analysis of neurite outgrowth, 20,000 cells isolated from postnatal day 1 (P1) mouse hippocampi were seeded into 96-well plates. For each pup, four wells of cells were seeded and analyzed, which served as a means of experimental replication. Plates were placed into the IncuCyte S3 Live-Cell Analysis System at 1 DIV, and neurite outgrowth was followed by automated continuous live-cell imaging. Four phase-contrast images per well were imaged at intervals of 6 h with a 10× objective lens for 15 DIV in total. For neurite outgrowth analysis, recorded images were analyzed using the automated NeuroTrack image acquisition module of the IncuCyte Zoom software. The NeuroTrack algorithm automatically defines cell bodies and neurite extensions (see Fig. 5*C*) to calculate and quantify the collective neurite length and neurite branch points per cell body cluster. Neurite length and neurite branch points were normalized to values for these parameters at time 0 h; data are presented as fold *x* values at time 0 h (time 0 h = 1).

### Statistical analysis

A summary of statistical analyses relating to this study is provided in Table 3. Two-tailed Student’s *t* tests were performed for all group comparisons. For neurite length and neurite branch point measures (see Fig. 5*A*,*B*), two-way ANOVA followed by Tukey’s multiple comparison tests were performed using GraphPad Prism 7 to compare the means for each genotype at each time point to one another. Data are presented as the mean ± SEM.

**Table 3:** Summary of statistical analyses

		WT			NHE6A11S			
Figure/statistical test/measure	Brain region or cells	Mean	SEM	*n*	Mean	SEM	*n*	Statistics
Figure 2/two-tailed Student’s *t* test/protein expression level								
	Whole brain	0.27 (70 kDa) 0.029 (140 kDa)	0.02 0.003	6 pups	0.27 (70 kDa) 0.023 (140 kDa)	0.02 0.004	4 pups	*p* = 0.455
Figure 3/two-tailed Student’s *t* test/Tfn subcellular localization								
	Cultured hippocampal neurons	294.4 Tfn endosomes (neurites)	41.8	5 pups 2723 cells 757,996 Tfn endosomes	305.9 Tfn endosomes (neurites)	25.4	5 pups 3147 cells 875,567 Tfn endosomes	*p* = 0.74
		49.2 Tfn endosomes (soma)	4.3	5 pups 2723 cells 132,822 Tfn endosomes	48.2 Tfn endosomes (soma)	3.0	5 pups 3147 cells 151,240 Tfn endosomes	*p* = 0.84
Figure 4/two-tailed Student’s *t* test/endosome pH								
	Cultured hippocampal neurons	6.48 (neurites)	0.04	9 pups 4310 cells 455,578 endosomes	6.45 (neurites)	0.06	8 pups 4525 cells 683,592 endosomes	*p* = 0.67
		6.21 (soma)	0.05	9 pups 4310 cells 47,809 endosomes	6.14 (soma)	0.06	8 pups 4525 cells 47,276 endosomes	*p* = 0.38
Figure 4/two-tailed Student’s *t* test/endosome area (μm^2^)								
	Cultured hippocampal neurons	1.034 (neurites)	0.01	9 pups 4310 cells 455,578 endosomes	1.025 (neurites)	0.009	8 pups 4525 cells 683,592 endosomes	*p* = 0.51
		0.925 (soma)	0.01	9 pups 4310 cells 47,809 endosomes	0.914 (soma)	0.02	8 pups 4525 cells 47,276 endosomes	*p* = 0.57
Figure 5/two-way ANOVA followed by Tukey’s multiple comparison test/neurite length								
	Cultured hippocampal neurons	See figure		4 pups (WT) 3 pups (NHE6-null)	See figure		11 pups (WT) 9 pups (NHE6A11S)	*p* < 0.0001 (WT vs. NHE6-null) *p* = 1 (WT vs. NHE6A11S)
Figure 5/two-way ANOVA followed by Tukey’s multiple comparison test/neurite branch points								
	Cultured hippocampal neurons	See figure		4 pups (WT) 3 pups (NHE6-null)	See figure		11 pups (WT) 9 pups (NHE6A11S)	*p* = 0.99 (WT vs. NHE6-null) *p* = 1 (WT vs. NHE6A11S)
Figure 6/two-tailed Student’s *t* test/area (cm^2^)								
	Cerebrum	0.97	0.01	5 pups	0.994	0.005	4 pups	*p* = 0.100
	Cerebellum	0.298	0.009	5 pups	0.306	0.008	4 pups	*p* = 0.535
	Cerebellum + Midbrain	0.348	0.009	5 pups	0.353	0.007	4 pups	*p* = 0.688
	Whole brain	1.31	0.02	5 pups	1.346	0.007	4 pups	*p* = 0.155
Figure 6/two-tailed Student’s *t* test/Purkinje cell density (cells/100 μm cell layer)								
	Cerebellum	5	0.2	4 pups	5	0.1	4 pups	*p* = 0.974

Tfn, Transferrin; WT, wild-type.

## Results

### Population frequency analysis of the A9S variant in SLC9A6

**Figure 1.**
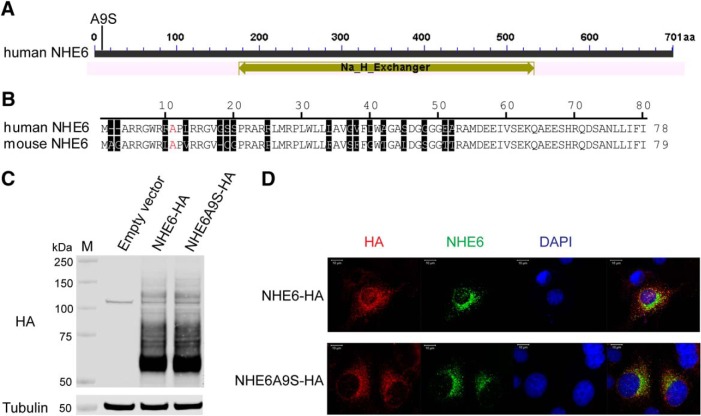
Evaluation of the NHE6A9S mutant in cultured cells. ***A***, Bar diagram of human NHE6. The predicted region of the conserved Na^+^/H^+^ exchanger domain (gold arrow) and the alanine-to-serine mutation at position 9 (A9S) are shown. ***B***, Alignment of amino acid sequences of human NHE6 and mouse NHE6 from the predicted N terminus through to the end of the second transmembrane domain. The alanine (A) at position 11 in mouse NHE6 corresponds to the alanine (A) at position 9 in human NHE6 (both in red font). Amino acids not conserved between the sequences or shifts in a sequence for optimal alignment are shaded. ***C***, Western blot of lysates from HeLa cells expressing HA-tagged human forms of wild-type NHE6 (NHE6-HA) or the NHE6A9S mutant (NHE6A9S-HA). Cells transfected with vector alone were used as a control. Lysates were probed with an anti-HA antibody to detect exogenously expressed NHE6 protein and an anti-tubulin antibody to detect tubulin, as a loading control. ***D***, Confocal microscopy images of HeLa cells expressing NHE6-HA or NHE6A9S-HA and immunostained for HA (red) and NHE6 (green). Nuclei were counterstained using DAPI (blue). Scale bars, 10 μm.

Fichou et al. (2009) identified a missense mutation (c.25G>T, p.A9S) in *SLC9A6* in a cohort of male patients from white or North-African origin with AS-like features (1 of 59). This mutation is localized in *SLC9A6* exon 1 and substitutes a polar amino acid, serine, for a nonpolar amino acid, alanine, at position 9 of the protein (Fig. 1*A*). The corresponding alanine in mouse NHE6 is at position 11 (Fig. 1*B*). It was proposed that alanine at position 9 (NP_006350.1) is highly conserved in several vertebrates, indicating that nonconservative mutation of this amino acid may alter the function of NHE6 (Fichou et al., 2009). To investigate whether the *SLC9A6* c.25G>T variant could be found in the general population, Fichou et al. (2009) screened 400 unaffected male individuals and identified 1 subject (of Egyptian origin) containing the variant. Combining the affected and unaffected male populations, the *SLC9A6* c.25G>T variant was suggested to be a rare polymorphism with an estimated frequency of 0.4% (2 of 459 individuals; Fichou et al., 2009).

The purpose of the study presented here was to investigate a larger control population in an effort to establish a better understanding of the genetic consequence of the c.25G>T variant of *SLC9A6*. Using data contained in the ExAC database (http://exac.broadinstitute.org/; Lek et al., 2016), 14,858 *SLC9A6* alleles were screened, and an allele count of 114 was determined with respect to the c.25G>T variant, resulting in an estimated frequency of 0.767% (114 of 14,858 alleles; Table 1). The sex skew of the NHE6A9S variant was then calculated based on the data reported in the ExAC database (Table 2). Using the values presented in Table 1, together with the equations shown in Materials and Methods, the observed and expected numbers of individuals with each possible genotype for the c.25 locus of *SLC9A6* were calculated. The results of these calculations are shown in Table 2. The results indicate that a greater number of males hemizygous for the NHE6A9S variant are observed than expected, whereas fewer females heterozygous for this variant are observed than expected. This suggests a lack of selective pressure against males hemizygous for the NHE6A9S variant. The predicted functional consequence of the NHE6A9S mutation was evaluated using PolyPhen-2 and SIFT. In using Polyphen-2, the NHE6A9S mutation is predicted to be benign. In using SIFT, however, this mutation is predicted to affect NHE6 function, although the number of sequences available for comparison was quite limited in using this program.

### *In vitr*o functional evaluation of the NHE6A9S mutation

To evaluate the effects of the NHE6A9S mutation on the function of NHE6 in an *in vitro* cellular model, the G > T substitution was introduced into the cDNA of wild-type human *SLC9A6* in a mammalian expression vector allowing for tagging of a protein at its C terminus with HA (NHE6-HA for wild-type NHE6; NHE6A9S-HA for mutant NHE6). The constructs encoding for NHE6-HA and NHE6A9S-HA were then expressed in HeLa cells, and protein expression was evaluated by Western blotting. Wild-type NHE6 typically migrates as multiple bands when cell or tissue lysates are separated using SDS-PAGE; these bands reflect the different glycosylated and oligomeric states of NHE6 (Ohgaki et al., 2008; Ilie et al., 2014). As presented in Figure 1*C*, HA-tagged NHE6A9S migrates as a smear of bands spanning from 60 to 150 kDa, similar to wild-type HA-tagged NHE6, with dominant bands at ∼60, 70, and 125 kDa. These results suggest that NHE6A9S undergoes appropriate glycosylation and oligomerization. The expression levels of wild-type NHE6-HA and NHE6A9S-HA were also similar, suggesting similar levels of protein stability.

Previous studies have shown that NHE6 is detected as puncta localized throughout the cellular cytoplasm and also in the perinuclear region, likely reflecting its localization to various endosomal populations and trafficking through the Golgi apparatus for post-translational modification (Brett et al., 2002; Nakamura et al., 2005; Ohgaki et al., 2008, 2010; Ouyang et al., 2013). Results from immunocytochemistry and confocal microscopy-based imaging presented here indicate that NHE6A9S-HA localizes in a similar distribution pattern (Fig. 1*D*). Thus, the alanine-to-serine mutation does not appear to affect the subcellular localization of NHE6. Together, the results from these *in vitro* studies in HeLa cells support that the A9S mutation does not affect the maturation, stability, or intracellular localization of NHE6.

### Protein stability and subcellular localization of NHE6A11S in a mouse model

**Figure 2.**
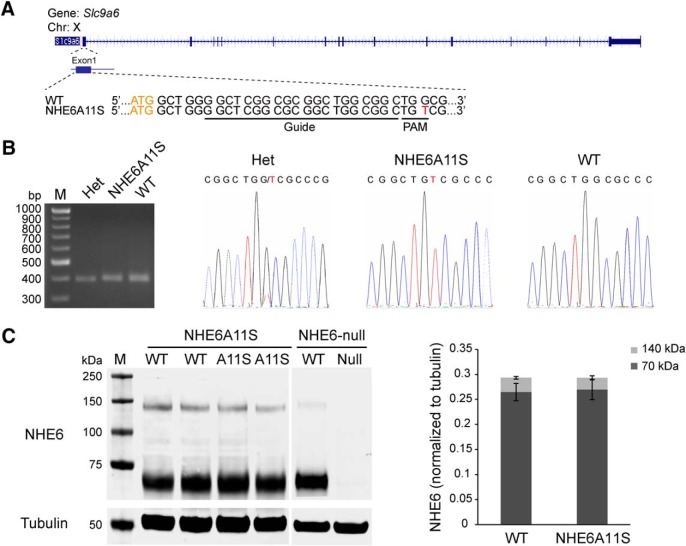
Validation and initial characterization of the NHE6A11S mouse line. ***A***, Diagrammatic representation of the targeted exon of mouse *Slc9a6* (*Nhe6*). The target sequence for CRISPR/Cas9-based genome editing is also shown [wild-type (WT), top; NHE6A11S, bottom]. The A11S point mutation (G > T) is in red font; the 20 nt sgRNA target sequence (Guide) and the protospacer adjacent motif (PAM) are underlined; and the start codon is in orange font. ***B***, Left, PCR genotyping gel showing a band of 402 bp present in littermate mice of genotypes heterozygous (het), NHE6A11S mutant, and WT. Right, Sanger sequencing chromatograms of the purified 402 bp PCR products confirming the presence of the G > T point mutation in samples from the heterozygous and NHE6A11S-mutant mouse lines. The mutant T nucleotide is in red font. ***C***, Left, Western blot of mouse brain lysates from WT and mutant mice of the newly generated NHE6A11S and *Nhe6*-null mouse lines. NHE6 is detected in samples from both WT and mutant mice for the NHE6A11S mouse line, whereas NHE6 is detectable only in the sample from WT mice for the *Nhe6*-null mouse line. Right, Quantification of Western blots. Quantifications were performed separately for bands at 70 kDa (dark gray) and 140 kDa (light gray). NHE6 expression was normalized to tubulin. Data are presented as the mean ± SEM. Statistical analyses were conducted using two-tailed Student’s *t* tests. No statistically significant difference in NHE6 levels was detected for samples from WT versus NHE6A11S-mutant mice.

To allow for analysis of the effects of the NHE6A9S variant on neuronal form and function and brain development, including *in vivo* analyses, a mouse model was generated using CRISPR/Cas9-mediated genome editing (Fig. 2*A*). In mouse NHE6, the corresponding alanine is in position 11 (Fig. 1*B*, amino acid sequence alignment). Thus, single guide RNAs (sgRNAs) were designed to introduce an A11S mutation into exon 1 of mouse *Slc9a6* (Fig. 2*A*). PCR-based genotyping together with Sanger sequencing showed successful G-to-T genome editing in NHE6A11S-mutant mice (Fig. 2*B*), indicating that the desired missense mutation had been generated in mouse *Slc9a6* using the CRISPR/Cas9 system. The assessment of NHE6 protein levels in mouse brain lysates by way of Western blotting supported that NHE6A11S-mutant protein, which is expressed from the endogenous *Slc9a6* locus, is expressed to an equivalent level, is equally stable, and undergoes similar post-translational processing as wild-type NHE6 (Fig. 2*C*, quantification on right). In contrast, NHE6 is undetectable in Western blotting of brain lysate from mice of a newly generated *Nhe6*-null line due to a 4 bp deletion in exon 15 that results in a frameshift and subsequent generation of a premature stop codon (Fig. 2*C*; see Materials and Methods).

**Figure 3.**
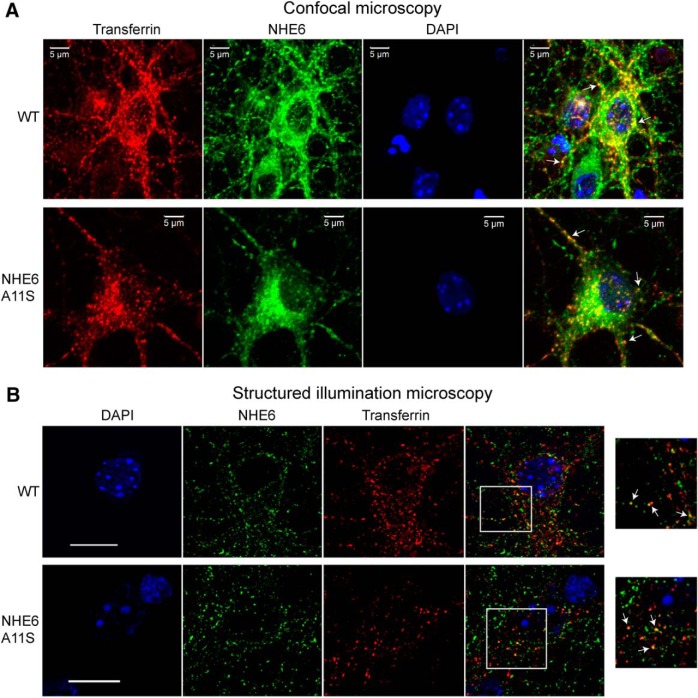
Distribution of NHE6A11S in mouse hippocampal neurons. ***A***, ***B***, Fluorescence microscopy images of dissociated hippocampal neurons from wild-type (WT) and NHE6A11S-mutant mice incubated with Alexa Fluor 568-conjugated transferrin (red) for 10 min to label early endosomes and immunostained for NHE6 (green). Nuclei were counterstained using DAPI (blue). Wide-field confocal microscopy images show colocalization (yellow) of NHE6 and transferrin at presumable early endosomes (arrows; ***A***). SIM images show areas of colocalization between NHE6 and transferrin (arrows), as well as areas of distinct, nonoverlapping puncta. The areas boxed in white are magnified to the right (***B***). Scale bars, 5 μm.

Next, the intracellular localization of NHE6A11S, compared with wild-type NHE6, was analyzed in hippocampal neurons isolated from mouse pups of the respective genotypes. As a marker for early endosomes, neurons were incubated with Alexa Fluor-conjugated transferrin for 10 min. For fixed-cell analysis, neurons were then stained for NHE6 using immunocytochemistry and imaged using confocal microscopy (Fig. 3*A*) as well as SIM (Fig. 3*B*). As shown in Figure 3, NHE6A11S displayed a characteristic vesicular distribution that overlapped with transferrin-positive compartments, a distribution pattern similar to that of wild-type NHE6 in neurons (Ouyang et al., 2013). To check whether the expression of the NHE6A11S variant changes transferrin distribution in neurons with respect to soma versus neurites, we quantified the number of transferrin-positive endosomes in each region using a high-throughput confocal microscope—the Opera Phenix High-Content Screening System (see Materials and Methods). Our results showed that there is no significant difference in the distribution of transferrin-positive endosomes to neuronal soma and neurites in wild-type versus NHE6A11S-mutant neurons. Calculated numbers of transferrin-positive endosomes in each region for each genotype were as follows (numbers per cell are indicated): neurite transferrin-positive endosomes, 294.4 ± 41.8 (wild-type), 305.9 ± 25.4 (NHE6A11S); soma transferrin-positive endosomes, 49.2 ± 4.3 (wild-type), 48.2 ± 3 (NHE6A11S); *p* = 0.74 for neurite transferrin-positive endosomes, *p* = 0.84 for soma transferrin-positive endosomes. Data were obtained from five animals of each genotype from two independent litters (Table 3). Overall, these results support that, in mouse neurons, the A11S point mutation of NHE6 does not affect its stability, maturation, or intracellular localization.

### Intraendosomal pH in neurons from the NHE6A11S mouse model

**Figure 4.**
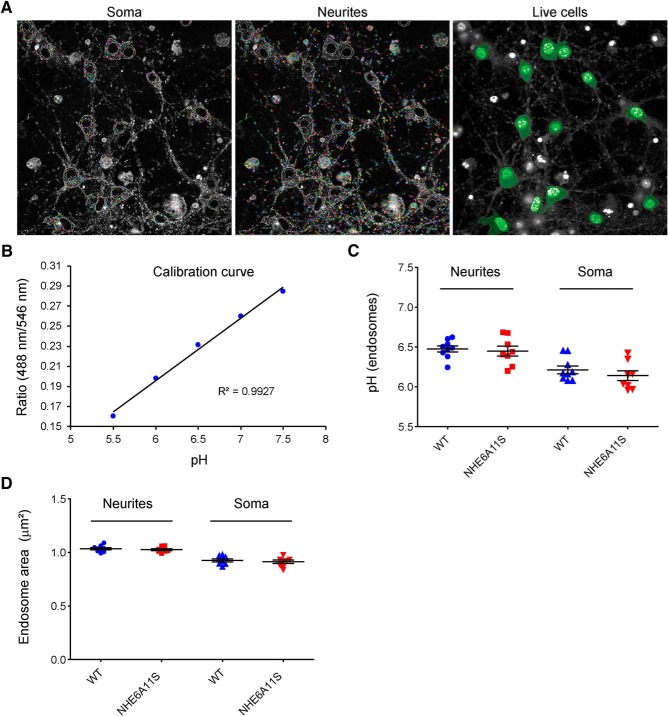
High-throughput confocal microscopy-based evaluation of the effects of NHE6A11S expression on endosomal pH and endosome area in mouse hippocampal neurons. ***A***, Opera Phenix High-Content Screening System images based on a single image of hippocampal neurons showing the fluorescence image and masking for identifying transferrin-labeled endosomes (rainbow color) in soma and neurites. Soma of the identified live cells are demarcated in green (right). Wild-type (WT) and NHE6A11S-mutant hippocampal neurons were loaded simultaneously with FITC-conjugated transferrin and Alexa Fluor 546-conjugated transferrin for 10 min and then imaged under live conditions in regular Neurobasal-A media, pH 7.4. ***B***, A pH calibration curve generated based on images such as those shown in ***A***. The wells for generating the pH calibration curve were incubated with buffers of the indicated pH values at the time of imaging. ***C***, Luminal pH values of transferrin-positive endosomes in WT and NHE6A11S-mutant hippocampal neurons determined using the calibration curve shown in ***B***. ***D***, Areas of transferrin-positive endosomes in WT and NHE6A11S-mutant hippocampal neurons. pH values and endosome areas were determined for two separate populations of endosomes, those localizing to neurites and those localizing to soma, as indicated. Data are presented as the mean ± SEM. Statistical analyses were conducted using two-tailed Student’s *t* tests.

NHE6 has been identified to play an important role in the regulation of endosomal pH (Orlowski and Grinstein, 2004; Ohgaki et al., 2011; Kondapalli et al., 2014). As such, the effects of the expression of NHE6A11S on intraendosomal pH was tested, here, specifically in neurons. The pH of early endosomes in wild-type and NHE6A11S-mutant neurons was measured using a technique involving endocytosis of pH-sensitive and pH-insensitive fluorescent conjugates of transferrin and ratiometric imaging using the Opera Phenix High-Content Screening System and Harmony software (Fig. 4; see also Materials and Methods). For experimental measures, hippocampal neurons (wild-type and NHE6A11S-mutant) were preloaded with FITC-conjugated (pH-sensitive) transferrin and Alexa Fluor 546-conjugated (pH-insensitive) transferrin for 10 min to label endosomes, followed by live imaging (Fig. 4*A*). A calibration curve was generated as well by imaging of hippocampal neurons preloaded with the fluorescent conjugates of transferrin and then incubated with buffers of known pH at the time of imaging (Fig. 4*B*). With respect to endosomes located in the processes of neurons, the pH of early endosomes in wild-type neurons was 6.48 ± 0.04, and in NHE6A11S-mutant neurons was 6.45 ± 0.06 (*n* = 4 litters, 9 pups, 4310 cells, 455,578 endosomes for wild-type; *n* = 4 litters, 8 pups, 4525 cells, 683,592 endosomes for NHE6A11S; *p* = 0.67). With respect to endosomes localized in the soma, the pH of endosomes in wild-type neurons was 6.21 ± 0.05, while the pH of endosomes in NHE6A11S-mutant neurons was 6.14 ± 0.06 (*n* = 4 litters, 9 pups, 4310 cells, 47,809 endosomes for wild-type; *n* = 4 litters, 8 pups, 4525 cells, 47,276 endosomes for NHE6A11S; *p* = 0.38; Fig. 4*C*). The endosome area was also analyzed for these same populations of endosomes, with the following results: neurite endosome area: 1.034 ± 0.01 μm^2^ (wild-type); 1.025 ± 0.009 μm^2^ (NHE6A11S); soma endosome area: 0.925 ± 0.01 μm^2^ (wild-type), 0.914 ± 0.02 μm^2^ (NHE6A11S); *p* = 0.51 for neurite endosomes, *p* = 0.57 for soma endosomes (Fig. 4*D*). These results indicate that the expression of NHE6A11S does not significantly change the intraendosomal pH or area of transferrin-labeled endosomes in hippocampal neurons.

### Brain development and aging in the NHE6A11S mouse model

**Figure 5.**
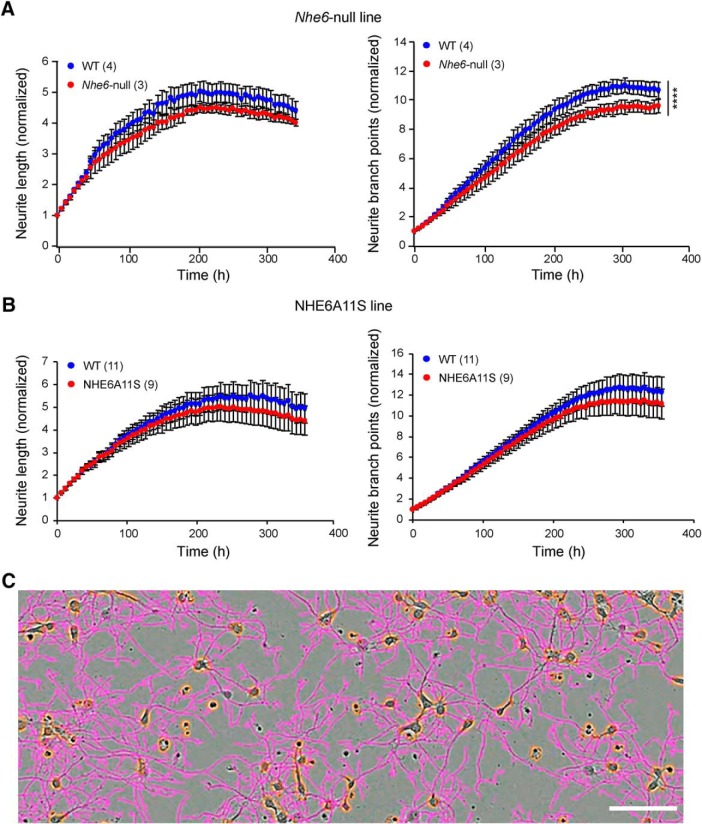
Live imaging-based evaluation of the effects of NHE6A11S expression on neuronal outgrowth. ***A***, ***B***, Measurement of neurite outgrowth with respect to neurite length (left) and branch points (right) in hippocampal neurons from an *Nhe6*-null mouse line (***A***) and the NHE6A11S mouse line (***B***). Wild-type (WT) and mutant neurons were analyzed for each line. Phase images were acquired from 1 DIV until ∼15 DIV. Neurite length and neurite branch points were normalized to values for these parameters at time 0 h; data are presented as fold *x* values at time 0 h (time 0 h = 1). Data are presented as the mean ± SEM. Statistical analyses were conducted using two-way ANOVA followed by Tukey’s multiple-comparison tests. ****p < 0.0001. ***C***, IncuCyte S3 Live-Cell Analysis System image from a single well of hippocampal neurons showing the phase image and masking for identifying neurites (purple) and cell bodies (orange). Scale bar, 100 μm.

NHE6 has been proposed to play roles in axonal and dendritic branching and in the formation of appropriate brain circuitry. Thus, whether the expression of NHE6A11S might affect related parameters, such as neurite extension and formation of neuronal branch points, was investigated (Fig. 5). For these studies, time-lapse microscopy was performed on cultured hippocampal neurons isolated from P1 pups of wild-type and NHE6A11S-mutant mice using the IncuCyte Live-Cell Imaging System. Using this automated imaging platform, real-time images were generated throughout the culturing of neurons for up to ∼15 DIV (Fig. 5*C*). Hippocampal cultures isolated from pups of the *Nhe6*-null mouse line were used as a control as well as for comparison with results obtained from the NHE6A11S mouse line. To this end, consistent with the previous finding of a neuronal arborization defect in *Nhe6*-null mice (Ouyang et al., 2013), the number of neuronal branch points was significantly reduced in *Nhe6*-null neurons compared with wild-type neurons (Fig. 5*A*). Similar defects in neuronal branch points were not, however, observed for NHE6A11S-mutant neurons (Fig. 5*B*), suggesting that the A11S mutation of NHE6 does not affect neurite branching. Neither mutant mouse line showed a defect in neurite length compared with the respective wild-type control (Fig. 5*A*,*B*).

**Figure 6.**
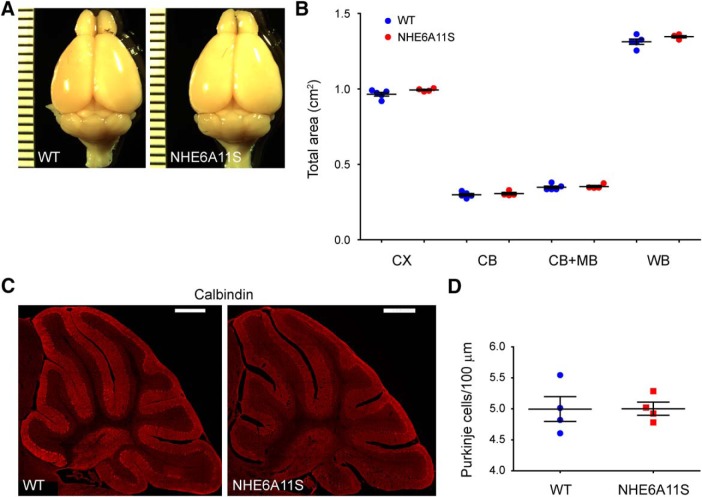
*In vivo* evaluation of the effects of NHE6A11S expression on mouse brain size and degeneration with aging. ***A***, Images of brains from wild-type (WT) and NHE6A11S-mutant mice at 6 months of age. ***B***, Quantitative analysis of brain size based on brains from mice at 6 months of age. CX, Cerebrum; CB, cerebellum; CB+MB, cerebellum + midbrain; WB, whole brain. ***C***, Confocal microscopy images of 30 μm sagittal sections of cerebella from WT and NHE6A11S-mutant mice at 6 months of age immunostained for calbindin, a marker specific to PCs. Scale bars, 500 μm. ***D***, Quantitative analysis of PC density based on cerebellar sections from mice at 6 months of age. Data are presented as the mean ± SEM. Statistical analyses were conducted using two-tailed Student’s *t* tests. No statistically significant differences in the sizes of the indicated brain regions (***B***) or PC density (***D***) were detected for samples from WT versus NHE6A11S-mutant mice.

In addition to neurodevelopmental defects caused by LoF mutations in *SLC9A6*, results suggesting neurodegenerative pathology, with age, in CS have been reported (Garbern et al., 2009, 2010; Strømme et al., 2011; Xu et al., 2017). In the mouse, *Nhe6*-null mice were found to have reductions in brain volume starting at 2 months of age, particularly in the cerebellum. Also, a progressive loss of PCs was observed in *Nhe6*-null mice, largely starting after 2 months of age and worsening over the course of a year (Strømme et al., 2011; Xu et al., 2017). Given these results reported for the *Nhe6*-null mouse line, the potential for a neurodegenerative phenotype in the NHE6A11S mouse line was analyzed. In performing direct measurement of gross brain size, no significant difference was detected between brains from wild-type mice and NHE6A11S-mutant mice at 6 months of age for all examined brain regions (cerebrum, cerebellum, cerebellum + midbrain, and whole brain; Fig. 6*A*,*B*). Degeneration of PCs also was not observed in analyzing cerebella from NHE6A11S-mutant mice at 6 months of age that had been immunostained for calbindin, a specific marker for PCs (Fig. 6*C*,*D*). These results suggest that brain of the NHE6A11S-mutant mouse does not undergo neuronal degeneration.

## Discussion

In the present study, a detailed examination of the molecular, cellular, and tissue consequences of a previously identified NHE6 missense variant, potentially causal of AS-like features in a male proband, was conducted. The missense mutation—NHE6A9S in human and NHE6A11S in mouse—substitutes a polar serine residue for a highly conserved, nonpolar alanine residue in the N terminus of NHE6. To perform this overall study, both exogenous expression (in cultured cells) and endogenous expression (in a mouse model) of mutant NHE6 were used and the effects analyzed. Compared with wild-type NHE6, exogenously expressed human NHE6A9S and endogenously expressed mouse NHE6A11S appeared to undergo normal protein synthesis, post-translational maturation, and dimerization. Mutant NHE6 protein also displayed a punctate localization pattern that overlapped with wild-type NHE6 and an endosomal marker, indicating that the alanine-to-serine mutation of NHE6 did not affect its ability to properly localize within cells. The expression of NHE6A11S also did not alter the distribution of transferrin-positive endosomes to neuronal soma versus neurites or appear to affect endosome area. Furthermore, unlike previous reports for *Nhe6*-null mice (Strømme et al., 2011; Ouyang et al., 2013; Xu et al., 2017), NHE6A11S-mutant mice did not show statistically significant defects in neurite branching or outgrowth, intraendosomal pH, brain size, or PC density. Together, the findings presented here indicate that the alanine-to-serine mutation (NHE6A9S or NHE6A11S) is not deleterious to the function of NHE6 with respect to these phenotypes.

As whole-genome sequencing and whole-exome sequencing approaches develop, more and more rare mutations are identified. To establish the functional consequences of variants identified in patients, experimental investigation of protein function may be helpful. Note, however, that the designation of a variant as pathogenic is a statistical argument based in human and population genetics (i.e., the presence of variant in affected individuals and the absence of variant in controls). The NHE6A9S variant was reported in a male with an AS-like phenotype but lacking an identified mutation in the relevant gene—*UBE3A*. The individual is reported as having many of the core symptoms of CS such as profound mental retardation, secondary microcephaly, hyperkinetic behavior, and absent speech. The possibility exists that a variant such as NHE6A9S may confer susceptibility to disease in a complex fashion. For example, a particular variant may not be necessary or sufficient on its own to cause disease but rather it may increase the statistical likelihood of symptom presentation through a model involving interactions with other genetic loci and/or the presence of specific environmental factors. This genetic model has yet to be tested in large case-control or family-based studies (i.e., transmission disequilibrium tests). Nonetheless, by screening a large control population in the ExAC database, a greater number of males hemizygous for the NHE6A9S variant were observed than expected, suggesting a lack of selective pressure against this variant in males. Thus, this analysis of population genetic data indicates that the NHE6A9S variant is not pathogenic in a highly penetrant, Mendelian fashion. This does not preclude the possibility that the A9S variant has a functional impact on NHE6, yet with incomplete penetrance; however, the failure to find a fewer number of observed males compared with expected males hemizygous for the NHE6A9S variant argues against this possibility. Combined, the data are suggestive of the concept that the NHE6A9S variant is not involved in disease phenotypes indicative of CS.

All assays for which results are reported here indicate that the NHE6A11S variant does not affect NHE6 function. LoF mutation of NHE6 causes aberrant overacidification of early endosomes. To measure intraendosomal pH, methods involving pH-sensitive fluorophore-conjugated transferrin are frequently used. We developed a high-throughput analysis method using the Opera Phenix High-Content Screening System. Using this method, we found that the A11S mutation of NHE6 did not affect intraendosomal pH. In a prior study, ∼83% of transferrin-positive early endosomes were found to costain with NHE6 (Ouyang et al., 2013). The formal possibility exists that NHE6 mutations could affect an endosome population not captured by this transferrin-based method of determining endosomal pH. However, the Opera Phenix High-Content Screening System allowed us to generate a large dataset through extremely sensitive confocal imaging and collect far more data points than could have been collected in the same amount of time by other reported methods, thereby documenting, with rigor, the absence of a phenotype in the transferrin-positive compartment.

In summary, the results reported here based in population genetics indicate that the NHE6A9S variant is unlikely to be pathogenic in a highly penetrant, Mendelian fashion. Results from mouse studies using a newly generated NHE6A11S mouse line are also supportive of this conclusion with respect to the phenotypes analyzed—neuronal arborization, intraendosomal pH, brain size, and PC density. More generally, the study presented here demonstrates a novel research pipeline for the evaluation of NHE6 variants found in CS patients, including the evaluation of the functional effects of variants *in vivo* when expressed under the endogenous promoter.

## Synthesis

Reviewing Editor: Lorna Role, NINDS Decisions are customarily a result of the Reviewing Editor and the peer reviewers coming together and discussing their recommendations until a consensus is reached. When revisions are invited, a fact-based synthesis statement explaining their decision and outlining what is needed to prepare a revision will be listed below.

In this manuscript, the authors explore the effect of a missense variant of uncertain significance in the SLC9A6 gene coding for the endosomal Na+/H+ exchanger NHE6. SLC9A6 mutations cause Christianson Syndrome, a severe X-linked neurodevelopmental disorder characterized by intellectual disability with non-verbal status, post-natal microcephaly, seizures, truncal ataxia, and hyperkinetic behavior, plus secondary motor regression and cerebellar atrophy. NHE6 controls endosomal luminal pH during endosome maturation from early to late endosomes. This is a critical process for intracellular trafficking and endosomal signaling. Here, the authors aimed to determine whether a p.A9S variant identified in humans had a deleterious effect on NHE6 function and brain development by studying it in both cell lines and a knock-in (KI) mouse model. The variant appeared to have no effect on protein stability and distribution and the neurons of the NHE6 KI animals had normal dendrite outgrowth and survival compared to NHE6 knock-out cells. The authors identified an increase in endosome acidification in the soma of KI neurons, but not in the dendrites, concluding that this variant only causes mild functional changes and may affect only a subset of endosomes. As the presentation of the individual with the p.A9S variant was more similar to Angelman Syndrome with only features of Christianson Syndrome, it is possible that additional variants may be contributing to the phenotype.

This is an elegant study, well-designed and well-controlled providing a comprehensive analysis of a possible disease variant using mouse KI approaches. There are a few concerns that the authors must address for publication, however.

MAJOR ISSUES

The reviewers were not fully convinced by the endosome acidification results. Both wanted a more thorough characterization of endosome distribution and transferrin colocalization in the transferrin experiments and felt that more detail - in particular, relative to the calculation of the n values - were needed to judge the robustness of the pH experiments.

1. RE: N values and figures chosen:

The authors must specify how many cells, how many different cultures from how many different animals were used for the determinations of transferrin (Fig 3) and for the assays of endosome acidification (Fig 4). Both reviewers felt that the number of endosomes tested was relatively small. These n values should be increased.

Specifically:

(a) The authors claim that there is no difference in transferrin distribution between WT and NHE6 KI neurons. However, from the image they chose to present in Figure 3 it appears that there is more transferrin in the recycling compartment than in the dendrites in NHE6 KI. While this apparent difference could just be the picture that was chosen, it would be important to provide a quantification of TF distribution in the soma and dendrites, particularly in light of the results on differential acidification in Figure 4.

(b) As discussed below, the partial colocalization of NHE6 and transferrin makes the issue of the n values and clarity of how these n were obtained of high importance. One can imagine that if the sampling is not sufficiently robust - i.e. across many cells, cultures and animals of WT vs NHE6 KI genotype- than the partial co-localization could skew the findings.

(c) For figure 4 please specify how many individual neurons were imaged for this set of experiments (from how many different WT and KI animals?) The authors only report the number of endosomes tested.

2. Use of Transferrin as endosome marker:

As transferrin and NHE6 only partially colocalize in vesicles, the vesicle pH distribution may be seriously influenced by pH of NHE6-negative vesicles. The choice of this marker should be justified and discussed.

Specifically:

Could FITC be directly coupled to NHE6 or via the HA tag to use, at least, in some of the in vitro experiments on vesicle pH? Such experiments are probably not going to affect the main conclusions of the manuscript, but the mean pH and standard deviation may be different also in dendrites. The authors should, at a minimum discuss the possible limitations of the assays used.

3. Effects on dendrites vs Soma:

There is no discussion on why the endosomes in the dendrites would be spared vs the soma. It is possible that the A9S/A11S variant may be only partially slowing down NHE6 activity and that the acidification phenotype is only observed in recycling and late endosomes. It is also possible that there may be differences in endosome distribution and trafficking. The additional quantification requested should provide some data to discuss this result in more detail.

ADDITIONAL POINTS

i. Choice of the Specific NHe6 mutation:

The rationale for picking the specific NHE6 mutation, where the human phenotype seems to be milder than full CS, should be discussed as this does increase the risk of obtaining the mainly negative findings reported here.

ii. Variance in WT cell lines

In figure 5, there is a larger difference in the data from wildtypes between cell lines (WT panel A vs. WT panel B) than between Nhe6-null and Nhe6A11S. Perhaps these differences between wildtypes masks for a phenotype of the mutation?

## Author Response

MAJOR ISSUES

The reviewers were not fully convinced by the endosome acidification results. Both wanted a more thorough characterization of endosome distribution and transferrin colocalization in the transferrin experiments and felt that more detail - in particular, relative to the calculation of the n values - were needed to judge the robustness of the pH experiments.

1. RE: N values and figures chosen:

The authors must specify how many cells, how many different cultures from how many different animals were used for the determinations of transferrin (Fig 3) and for the assays of endosome acidification (Fig 4). Both reviewers felt that the number of endosomes tested was relatively small. These n values should be increased.

Response: To address this question, we developed a method using a high-throughput confocal fluorescence imaging system, the Opera Phenix High Content Screening System, which allowed us to image and analyze thousands of cells and endosomes. Based on use of this new method and imaging system, we have added the n values for data in Fig. 3 and Fig. 4 in the text and Table 3, including with respect to litters, pups, cells, and endosomes.

Specifically:

(a) The authors claim that there is no difference in transferrin distribution between WT and NHE6 KI neurons. However, from the image they chose to present in Figure 3 it appears that there is more transferrin in the recycling compartment than in the dendrites in NHE6 KI. While this apparent difference could just be the picture that was chosen, it would be important to provide a quantification of TF distribution in the soma and dendrites, particularly in light of the results on differential acidification in Figure 4.

Response: Again using the Opera Phenix High Content Screening System, we were able to image thousands of cells and quantify transferrin distribution in neuronal soma and neurites. The result showed that there is no significant difference in transferrin distribution to soma and neurites in hippocampal neurons from wild-type vs. NHE6A11S-mutant mice. Detailed information about the method has been added to the Materials and Methods and the Results have been revised to include these data. Also, the text and Table 3 has been revised to include the n values for these analyses.

(b) As discussed below, the partial colocalization of NHE6 and transferrin makes the issue of the n values and clarity of how these n were obtained of high importance. One can imagine that if the sampling is not sufficiently robust - i.e. across many cells, cultures and animals of WT vs NHE6 KI genotype- than the partial co-localization could skew the findings.

Response: We have now included details of n values for our analyses regarding data in Fig. 3 and Fig. 4 in the text (Materials and Methods, and Results) and Table 3.

(c) For figure 4 please specify how many individual neurons were imaged for this set of experiments (from how many different WT and KI animals?) The authors only report the number of endosomes tested.

Response: We have now included details of n values for our analyses regarding data in Fig. 4 in the text (Materials and Methods, and Results) and Table 3.

2. Use of Transferrin as endosome marker:

As transferrin and NHE6 only partially colocalize in vesicles, the vesicle pH distribution may be seriously influenced by pH of NHE6-negative vesicles. The choice of this marker should be justified and discussed.

Response: We have now added text concerning the co-localization between NHE6 and transferrin and use of the fluorescently-conjugated transferrin-based method for measuring endosomal pH to the Discussion. This includes a discussion of use of this method in combination with the Opera Phenix High Content Screening System.

Specifically:

Could FITC be directly coupled to NHE6 or via the HA tag to use, at least, in some of the in vitro experiments on vesicle pH? Such experiments are probably not going to affect the main conclusions of the manuscript, but the mean pH and standard deviation may be different also in dendrites. The authors should, at a minimum discuss the possible limitations of the assays used.

Response: We have now revised the Discussion to include text concerning potential limitations of using the transferrin-based method to determine whether expression of the NHE6A11S mutant might affect endosomal pH, given the lack of 100% co-localization between NHE6 and transferrin.

3. Effects on dendrites vs Soma:

There is no discussion on why the endosomes in the dendrites would be spared vs the soma. It is possible that the A9S/A11S variant may be only partially slowing down NHE6 activity and that the acidification phenotype is only observed in recycling and late endosomes. It is also possible that there may be differences in endosome distribution and trafficking. The additional quantification requested should provide some data to discuss this result in more detail.

Response: In using the Opera Phenix High Content Screening System and performing additional quantification, we now do not detect a statistically significant difference in endosomal pH in neuronal soma or dendrites between wild-type vs. NHE6A11S-mutant hippocampal neurons. The previously reported slight, but statistically significant, reduction in endosomal pH in somal endosomes only in NHE6A11S-mutant neurons in comparison to wild-type neurons may have been due to the partial colocalization of NHE6 and transferrin and/or the n values at that time.

ADDITIONAL POINTS

i. Choice of the Specific NHe6 mutation:

The rationale for picking the specific NHE6 mutation, where the human phenotype seems to be milder than full CS, should be discussed as this does increase the risk of obtaining the mainly negative findings reported here.

Response: Text concerning the basis for this study is provided at the conclusion of the Introduction (final paragraph) and the beginning of the Discussion (portions of first two paragraphs).

ii. Variance in WT cell lines

In figure 5, there is a larger difference in the data from wildtypes between cell lines (WT panel A vs. WT panel B) than between Nhe6-null and Nhe6A11S. Perhaps these differences between wildtypes masks for a phenotype of the mutation?

Response: This difference between wild-type animals of the two different mouse lines may be due to the different genetic backgrounds of these lines C57BL/6J for the Nhe6-null line in our initially submitted manuscript and C57BL/6N for the NHE6A11S line. To address this question, we have repeated the analyses shown in Fig. 5 using an Nhe6-null mouse line we have newly generated that is on the C57BL/6N background. Under the same genetic background, the variance between the respective wild-type animals for these two lines is reduced, with the wild-type animals showing similar increases in neurite length and neurite branch points over time. The Materials and Methods, Fig. 2 and Fig. 5, and figure legends have been revised to describe this new Nhe6-null mouse line and include updated data.
